# Agricultural Injuries With Dementia: Double Whammy?

**DOI:** 10.1002/ajim.70082

**Published:** 2026-04-16

**Authors:** Kanika Arora, Jonathan Davis, Lila B. Basnet, Julie Bobitt

**Affiliations:** ^1^ Department of Health Management and Policy University of Iowa Iowa City Iowa USA; ^2^ Department of Occupational and Environmental Health University of Iowa Iowa City Iowa USA; ^3^ Department of Medicine, Center for Dissemination and Implementation Science University of Illinois Chicago Chicago Illinois USA

**Keywords:** agriculture, dementia, injury, rural

## Abstract

**Background:**

Nearly 40% of US farmers are over 65 years old. Some emerging evidence links agricultural occupational exposure to increased dementia risk. However, little is known about dementia and injury outcomes in agricultural settings.

**Methods:**

We employed data from the American College of Surgeons Trauma Quality Programs Participant Use File (ACS‐TQP‐PUF) (2017–2021), identifying individuals aged ≥ 60 with (1) agricultural injuries and dementia (Group 1; *N* = 318), (2) agricultural injuries without dementia (Group 2; *N* = 21,361), and (3) dementia without agricultural injuries (Group 3; *N* = 231,231). Injury mechanisms were compared across groups using chi‐square tests. Injury severity was assessed via Injury Severity Score and the use of intensive care unit (ICU) or surgical care by hospitalized patients. Generalized ordered logit and logistic regression models estimated associations between group membership and injury severity, adjusting for demographics and comorbidities.

**Results:**

Falls caused 94% of traumatic injuries in individuals with dementia alone but accounted for only 35% of injuries in those with both agricultural injuries and dementia. Group 1 and Group 2 experienced a broader range of injury mechanisms including motor vehicle traffic, transport, and environmental incidents. Compared to Group 3, Group 1 had higher probability of experiencing major injuries and receiving ICU/surgical care. Injury severity was largely similar between Groups 1 and 2.

**Conclusions:**

This cross‐sectional study provides suggestive evidence that older adults with dementia and agricultural injuries experience more severe outcomes than those in nonagricultural settings. Future research should explore this group in greater depth as well as the implications for dementia caregivers in agricultural communities.

## 1 Introduction

With a fatality rate more than five times the national average, agriculture ranks among the most hazardous US industries [1]. Unlike other occupations, farming lacks a defined retirement age, with nearly 40% of producers aged 65 years or older (a 12% increase since 2017) [2]. Older farmers face disproportionately high rates of mortality and morbidity, accounting for about 80% of all farm‐related fatalities and nearly a quarter of all agriculture‐related emergency department (ED) visits [3, 4].

Advanced age is the primary risk factor for dementia [5]—an umbrella term for a group of neurodegenerative disorders that cause devastating impacts to health. Moreover, some evidence from the United States and Europe indicates a potential relationship between agricultural work (or exposures related to agricultural work) and an increased risk for cognitive impairment and dementia even after accounting for age. One study employed nationally representative data from the United States and found that older Americans with longest‐held jobs in agriculture experienced 46% greater odds of developing dementia relative to socio‐demographically similar peers in other occupations [6]. Results also showed that although agricultural workers started with lower cognitive scores (i.e., experienced early onset), nonagricultural workers exhibited faster cognitive declines over time, possibly due to greater accumulation of brain pathology (as predicted by the cognitive reserve framework) [6]. In another case‐control study, occupational pesticide exposure among older residents of an agricultural region of Cache County, Utah, was associated with increased risks for developing all‐cause dementia as well as Alzheimer's disease (AD)—the most common type of dementia [7]. These findings were corroborated in a recent French study where authors examined the association between different types of agricultural activities and the risk of AD using nationwide health insurance data of French producers [8]. Specifically, researchers analyzed 26 distinct types of agricultural activities and showed higher risk of AD among producers whose activities involved frequent use of pesticides (crop farming, viticulture, and fruit arboriculture). In contrast, several animal farming activities (e.g., poultry and rabbit farming) were associated with lower AD risk. A handful of older studies from Europe show mixed findings. In a longitudinal study of community‐dwelling Spanish elderly with low‐levels of formal education and predominantly unskilled occupations, being a farmworker was associated with deterioration in cognition over a 4‐year period [9]. Similarly, in another study of older community residents from Bordeaux, France (PAQUID cohort), being a farmworker or a farm manager was associated with a higher risk of cognitive impairment even after controlling for age and education levels [10, 11]. However, in a follow‐up study of the PAQUID cohort—that went beyond predicting cognition and examined the risk of incident dementia—researchers were unable to detect an association between lifetime occupation and the risk of AD [12]. At the same time, this study did show that being a farmer was associated with an increased risk of dementia with parkinsonism, particularly among women [12].

Although evidence on the association between agricultural work and dementia has yet to reach a consensus, examining the effects of dementia among farmers—who are aging rapidly and often continue to work in hazardous settings well into advanced age—is both significant and timely as dementia can potentially amplify the risk of workplace injuries. Slow‐progressing initial symptoms, such as poor judgment, visual‐spatial deficits, and balance problems, often go unrecognized [13] or are sometimes dismissed as normal aging [14], particularly in rural areas where the disease is frequently underdiagnosed [15]. These symptoms can impair an individual's ability to safely navigate their surroundings, increasing the risk of injury and related hospitalization [16]. Safety concerns associated with dementia are amplified in farm environments, which present numerous hazards including those from heavy equipment, livestock, confined spaces (e.g., grain bins and manure pits), and firearms. This risk is compounded by older farmers' lower likelihood of using protective equipment [17]. Given the interdependent nature of agricultural work, dementia in an older farm worker can increase safety risk for other workers as well. Finally, because many farms blend work and living areas, dementia‐related behaviors such as wandering can also present safety risks even for those who simply reside on a farm.

Although older farmers continue working and living on family farms well into late adulthood [18], no prior study has examined the link between dementia and agricultural injuries. This is in part because only a limited number of secondary datasets include information on the two central elements required for this analysis: the nature and type of injury (i.e., whether an injury was agriculture‐related) and the presence or absence of dementia. In general, current efforts to describe injuries among individuals with dementia have largely focused on falls and those that occur in non‐occupational settings [16, 19]. As such, there is a significant knowledge gap regarding whether dementia in older farm residents and agricultural workers is linked to different injury risks compared to other older adults. The purpose of this study was to address this research gap by analyzing national trauma data from the Trauma Quality Programs Participant Use File (TQP‐PUF). Specifically, we examined whether injury mechanisms and severity differed by dementia status and presence of agricultural injuries.

## 2 Methods

### 2.1 Data Source and Sample

This retrospective cohort study used data from the American College of Surgeons (ACS) TQP‐PUF for admission years 2017–2021. The ACS‐TQP‐PUF is a publicly available, validated, de‐identified, incident‐based dataset maintained by the ACS Committee on Trauma, containing information on trauma patients treated at over 700 trauma facilities across the United States, including Level I–V and undesignated trauma centers [20]. Each year, the study sample included patients aged 60 or older with a documented dementia diagnosis or a confirmed or suspected agricultural injury. Documented dementia was identified if it was (1) listed as a pre‐existing condition by the trauma center, or (2) recorded as a principal diagnosis during the ED visit (*International Classification of Diseases, Tenth Revision* [ICD‐10] codes for identifying dementia cases are listed in Supporting Information: Table S1). Confirmed or suspected agricultural injuries were identified using a previously published crosswalk between ICD‐10‐Clinical Modification (ICD‐10‐CM) external cause codes and the Bureau of Labor Statistics Occupational Injury and Illness Classification System (OIICS) [21].

Three subgroups of older adults were constructed: those with agricultural injuries and a dementia diagnosis (Group 1, *N* = 318), those with agricultural injuries but no dementia diagnosis (Group 2, *N* = 21,361), and those with a dementia diagnosis but no agricultural injuries (Group 3, *N* = 231,231).

### 2.2 Measures (Injury Mechanisms, Severity, and Covariates)

We determined each patient's injury mechanism using ICD‐10‐CM external cause codes, which the ACS‐TQP‐PUF maps to standardized categories from the National Center for Health Statistics [22]. We focused on six mechanisms, of which five—falls, motor vehicle traffic, other transport, machinery, natural/environmental, and struck by/against—accounted for ≥ 2% of injuries in at least one subgroup. All remaining mechanisms were coded as “other.” Injury severity was assessed two ways: (1) using Injury Severity Score (ISS) to classify injuries as minor (1–8), moderate (9–15), or major (> 15); and (2) among ED patients discharged to the hospital, by comparing ICU/surgical unit admissions (coded “1”) to general/floor bed, telemetry/step‐down, or observation unit admissions (coded “0”). In multivariate models, we adjusted for age category, sex, race, ethnicity, smoking status, and chronic conditions associated with both, dementia as well as related injuries, including diabetes, hypertension, congestive heart failure (CHF), myocardial infarction, and Chronic Obstructive Pulmonary Disorder (COPD) [23, 24]. We also controlled for the ACS‐TQP‐PUF data year.

### 2.3 Statistical Analysis

We used chi‐square tests to compare mechanisms of injury between Group 1 and Group 2, and between Group 1 and Group 3. Because of the ordinal nature of ISS‐based injury severity (minor, moderate, and major), we initially considered an ordered logistic model. However, due to a violation of the proportional odds assumption (indicated by a statistically significant test statistic for Brant test of the parallel regression assumption), we employed the generalized ordered logit model [25]. The generalized ordered logit model provides a less restrictive alternative to the proportional odds model, while remaining more parsimonious and interpretable than non‐ordinal methods [25]. We fit two separate, multivariate generalized ordered logit models to estimate the association between group membership and ISS‐based injury severity. Each model compared Group 1 to Group 2, and Group 1 to Group 3. In addition, we fit separate logistic regression models to examine the association between group membership and the likelihood of using ICU or surgical unit among patients discharged to the hospital, comparing Group 1 to Group 2, and Group 1 to Group 3. We also include injury mechanisms as covariates to account for confounding in the relationship between group membership and injury severity. Because agricultural injuries are more likely to involve inherently more severe mechanisms (such as equipment‐ or motor vehicle‐related incidents), any observed association between group membership and injury severity (particularly in Group 1 vs. Group 3) may essentially be capturing these differences. Additionally, we conduct mechanism‐stratified analyses to further assess this question. Outcomes are presented as average adjusted predictions, with *p*‐values (and associated confidence intervals) for group differences in outcomes derived from average marginal effects.

We conducted multiple sensitivity analyses. First, we restricted the sample to only true/confirmed agricultural injuries, excluding suspected cases [21]. Based on confirmed agricultural injury classification, sample sizes for the three subgroups were as follows: those with a confirmed agricultural injury and a dementia diagnosis (Group 1), *N* = 188; those with a confirmed agricultural injury but no dementia diagnosis (Group 2), *N* = 12,797; and those with a dementia diagnosis but no confirmed agricultural injury (Group 3), *N* = 231,361. Second, to enhance comparability between Groups 1 and 3, we redefined Group 3 by excluding injuries occurring in private or institutional residences, limiting the group to external/non‐agricultural incidents (*N* = 28,571). Finally, we also controlled for use of emergency medical services (EMS) transport as a covariate in multivariate analyses. Not only are farmers disproportionately located in rural areas where EMS utilization is generally lower [26], prior work also shows that they may be less likely to arrive by ambulance than other patients presenting to rural hospitals due to the unique nature of farmwork (e.g., unsupervised for long periods of time, imprecise location description, hazards at the scene such as livestock and machinery) [27, 28]. This may lead to worse injury outcomes as farmers may miss the benefit of formal triage by an EMS provider [28]. To mitigate potential bias from this confounder, we include a binary indicator coded as “1” if the individual was transported by ground, helicopter, or fixed‐wing ambulance, and “0” if the mode of transport was private vehicle, walk‐in, or police. This study used de‐identified data under a data use agreement (DUA) that prohibits re‐identification. Therefore, it was not considered human subjects research. The DUA procedures were reviewed and approved by the Institutional Review Board at the University of Iowa.

## 3 Results

### 3.1 Overall Descriptives

Table 1 summarizes characteristics of the three groups. Compared to individuals with only agricultural injuries (Group 2), those with both dementia and agricultural injuries (Group 1) were more likely to be over 80 years old and have diabetes, hypertension, CHF, and COPD. Groups 1 and 2 were largely similar in terms of sex, race, ethnicity, and use of EMS transport. Meanwhile when compared to individuals with a dementia diagnosis but no agricultural injuries (Group 3), Group 1 had fewer females, a higher percentage of white individuals, and lower likelihood of using EMS transport, but was similar in age, smoking habits, and cardiovascular comorbidities. There were no missing data in these variables for Group 1. EMS use was missing for fewer than 1% of cases in Groups 2 and 3. Race was missing for less than 1% of individuals in Group 3. These cases were dropped from the analysis.

**Table 1:** Characteristics of trauma patients (≥ 60 y) by dementia status and presence of agricultural injury.

	Dementia and ag. injury (Group 1) (*n* = 318)	Only ag. injury (no dementia) (Group 2) (*n* = 21,361)	Only dementia (no ag. injury) (Group 3) (*n* = 231,231)
Demographic characteristics (col %)			
Mean age in years (standard deviation)	78.2 (7.0)	70.1 (7.4)	81.0 (6.4)
Age 60–69	12.20%	52.50%	6.60%
Age 70–79	38.60%	34.40%	28.40%
Age 80+	49.10%	13.00%	65.00%
Female sex	29.50%	26.10%	62.60%
Race: White	96.20%	94.20%	86.50%
Hispanic	.	3.50%	5.80%
Health and healthcare use (col %)			
Current smoker	3.80%	10.00%	5.40%
Diabetes	25.80%	18.40%	24.60%
Hypertension	64.20%	51.10%	67.90%
CHF	11.00%	3.50%	10.70%
Myocardial infarction	.	1.20%	1.40%
COPD	14.00%	7.30%	12.80%
Use of Emergency Medical Services (EMS) Transport [ground, helicopter, and fixed‐wing]	86.44%	84.43%	91.02%

*Note:* Data come from the 2017–2021 American College of Surgeons Trauma Quality Programs Participant Use File (ACS‐TQP‐PUF). Missing (.) indicates suppressed values because cell count was < 11.

Abbreviations: Ag., agricultural; CHF, congestive heart failure; COPD, chronic obstructive pulmonary disorder.

### 3.2 Mechanism of Injury

In Table 2 we compare injury mechanisms across the three groups. Falls were the leading cause of injury in Groups 1 and 3, but their prevalence differed significantly—35% in Group 1 versus 94% in Group 3 (*p* < 0.001). In Group 2, falls accounted for only 22% of injuries, with transport (29%) and environmental factors (21%) encompassing other common mechanisms. Group 1 had a higher proportion of motor vehicle injuries compared to both Groups 2 (*p* = 0.07) and 3 (*p* < 0.001). Meanwhile, machinery and environmental injuries were more common in Group 2. Overall, aside from falls, Groups 1 and 2 experienced relatively comparable injury causes and a broader range than Group 3. Injury mechanisms were missing for less than 1% of the sample in all three groups. These cases were dropped from the analysis.

**Table 2:** Mechanism of injury in trauma patients (≥ 60 y) by dementia status and presence of agricultural injury.

	Dementia and ag. injury (Group 1) (*n* = 318)	Only ag. injury (no dementia) (Group 2) (*n* = 21,361)	Only dementia (no ag. injury) (Group 3) (*n* = 231,231)	*p*‐Value (Group 1 vs. Group 2)	*p*‐Value (Group 1 vs. Group 3)
Injury mechanism (Col %)					
Falls	35.30%	21.80%	94.47%	< 0.001	< 0.001
MVT	15.77%	12.40%	2.86%	0.07	< 0.001
Transport	26.50%	29.18%	0.06%	0.29	< 0.001
Machinery	3.47%	7.08%	0.02%	0.01	< 0.001
Environment	10.09%	20.68%	0.30%	< 0.001	< 0.001
Struck by/against	4.10%	4.00%	1.03%	0.94	< 0.001
Other	4.73%	4.87%	1.26%	0.91	< 0.001

*Note: D*ata come from the 2017–2021 American College of Surgeons Trauma Quality Programs Participant Use File (ACS‐TQP‐PUF). *p*‐Values are based on separate chi‐square tests comparing Group 1 with Groups 2 and 3. “Motor Vehicle Traffic (MVT)” includes MVT Occupant, MVT Motorcyclist, MVT Pedal cyclist, MVT Pedestrian, MVT Unspecified and MVT Other. “Environment” includes Natural/Environmental ‐ Bites and stings and Natural/Environmental ‐ Other. “Other” includes cut/pierce, fire/flame/hot objects, drowning/submersion, firearm, pedal cyclist/other, pedestrian/other, overexertion, poisoning, suffocation, other, unspecified, adverse effects/medical care, and adverse effects/drugs. Missing (.) indicates suppressed values because cell count was < 11.

Abbreviation: Ag., agricultural.

### 3.3 Injury Severity

#### 3.3.1 Descriptive Results

In unadjusted analyses (Supporting information: Table S2), compared to Groups 2 and 3, Group 1 was less likely to sustain minor injuries. ISS was missing for fewer than 1% of cases in Groups 2 and 3 and these were dropped from the analysis. There were no cases with missing ISS in Group 1. About 89% of Group 1 members were hospitalized after the ED visit, versus 87% in Group 2 and 91% in Group 3. However, a higher percentage of Group 1 was admitted directly to the trauma center. ED discharge disposition data is unavailable for these cases (6% direct admits in Group 1 vs. 4% direct admits in Groups 2 and 3) and they are excluded from the analysis. Overall, 36% of Group 1 required ICU or surgical intervention, compared to 34% in Group 2 and 23% in Group 3.

#### 3.3.2 Multivariate Results

**Figure 1:**
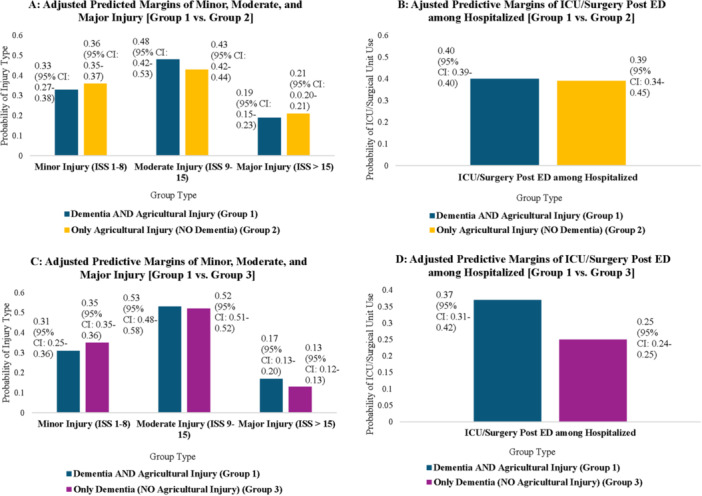
This figure presents adjusted predictive margins from the covariate‐adjusted generalized ordered logit (A and C) and logistic (B and D) regression models estimating ISS‐based injury severity and type of hospital care (respectively), comparing Group 1 with Groups 2 (A and B) and 3 (C and D). Data come from the 2017–2021 American College of Surgeons Trauma Quality Programs Participant Use File (ACS‐TQP‐PUF) and are restricted to patients ≥ 60 years in each year. Regressions were adjusted for age categories, female, black, other, Hispanic, smoking status, comorbidities (diabetes, hypertension, congestive heart failure, myocardial infarction, and chronic obstructive pulmonary disorder), and year fixed effects. For average marginal effects and associated *p‐*values for group differences in outcomes see Supporting information: Table S3 [A–D].

Figure 1 presents adjusted predictive margins from the covariate‐adjusted generalized ordered and logistic regression models estimating ISS‐based injury severity and type of hospital care (respectively), comparing Group 1 with Groups 2 and 3. These adjusted estimates closely mirror the unadjusted results. In terms of ISS‐based injury severity, 17% of Group 1 members were likely to sustain major injuries compared to 13% in Group 3 (Figure 1, Panel C) (*p* < 0.05 [95% confidence interval (CI): 0.001–0.06]; Supporting information: Table S3C). While Group 1 members were less likely to experience minor injuries compared to both Groups 2 (Figure 1A; *p* = 0.27 [95% CI: –0.09 to 0.02]; Supporting information: Table S3A) and 3 (Figure 1C; *p* = 0.07 [95% CI: –0.10 to 0.005]; Supporting information: Table S3C), and more likely to experience moderate injuries compared to Group 2 (Figure 1A; *p* = 0.11 [95% CI: –0.02 to 0.11]; Supporting information: Table S3A), these results were not statistically significant at the 5% level. A higher proportion of hospitalized Group 1 members received ICU care or surgical intervention compared to Group 3 (37% vs. 25%; *p* < 0.001) [95% CI: 0.06–0.17] (Figure 1D) (Supporting information: Table S3D). In contrast, there was no statistically significant difference in the likelihood of receiving such care between hospitalized Group 1 and Group 2 patients (Figure 1B; *p* = 0.92 [95% CI: –0.05 to 0.06]; Supporting information: Table S3B).

In additional analyses, binary variables for injury mechanisms (falls, motor vehicle traffic, transport, machinery, environment, struck by/against) were included as covariates in regressions predicting ISS‐based injury severity and receipt of ICU care or surgical intervention. Relative to Group 3, Group 1 was less likely to experience a minor injury (*p* = 0.01 [95% CI: –0.14 to –0.02]; Supporting information: Table S3E) and more likely to experience a moderate injury (*p* = 0.005 [95% CI: 0.02–0.14]; Supporting information: Table S3E). There was no statistically significant difference in the likelihood of experiencing a major injury between Group 1 and Group 3 (*p* = 0.78 [95% CI: –0.04 to 0.03]; Supporting information: Table S3E). Upon inclusion of injury mechanisms, the previously observed statistical significance for higher likelihood of ICU admission/surgical intervention in Group 1 relative to Group 3 was no longer detectable (*p* = 0.13 [95% CI: –0.01 to 0.09]; Supporting information: Table S3F). The inclusion of these additional covariates did not substantially alter the main findings when comparing Groups 1 and 2. For Groups 1 and 3, we also conducted stratified analysis for those injured by falls as this was the leading cause of injury in both groups. We find no statistically significant differences in injury severity between members of Groups 1 and 3 who were injured due to a fall (Supporting information: Table S3G and Table 3H).

#### 3.3.3 Sensitivity Analyses

These findings were robust to restricting the sample to true/confirmed agricultural injuries only, redefining Group 3 to exclude residential injuries, and including the use of EMS transport as an additional covariate. Findings from all sensitivity models are presented in Supporting information: Tables S4, S5, and S6 respectively. Across all three analyses, Group 1 experienced more severe outcomes than Group 3, as indicated by ISS‐defined injury categories and nature of hospitalization. In contrast, Group 1 and 2 experienced similar outcomes for the most part. For the third sensitivity check where EMS use was included as a covariate, relative to Group 2, Group 1 was slightly more likely to experience a moderate injury as well as more likely to receive ICU care/surgical intervention—these coefficients were statistically significant at the 10% level (Supporting information: Tables S6A and S6B).

## 4 Discussion

Employing a unique crosswalk that enables identification of work‐related injuries in hospital data, this is the first study to examine injury outcomes in individuals with both dementia and agricultural injuries. We find that compared to those with dementia alone (Group 3), the group with both agricultural injuries and diagnosed dementia (Group 1) experienced a broader range of injury mechanisms and greater injury severity, as reflected by ISS scores and nature of hospitalization. The higher severity in Group 1 compared to Group 3 likely reflects differences in injury mechanisms between the two groups. Non‐fall mechanisms on the farm may be inherently more severe. Indeed, when injury mechanisms were included as covariates in multivariate analyses, the difference in the probability of receiving ICU care or surgical intervention between Groups 1 and 3 was small and no longer statistically significant. Among individuals in Groups 1 and 3 injured due to falls, there was no statistically significant association between group membership and injury severity, further indicating that the main findings are likely driven by Group 1's greater exposure to more severe (nonfall) injury mechanisms. It is also important to note that ACS‐TQP‐PUF data are derived from trauma centers that voluntarily participate in TQP, which may not fully represent the entire landscape of agricultural injuries across the United States. This is particularly relevant if Level I and II trauma centers—typically larger and better resourced—are overrepresented in the data, while rural patients, who are more likely to be treated at smaller centers are underrepresented. If Level I and Level II trauma centers are overrepresented in our data, it is possible that Groups 1 and 2—that are predominantly rural—consist of more severe injury cases relative to Group 3. This selection bias could also be a potential driver of differences in injury severity between Groups 1 and 3.

Injury severity was generally similar between individuals with agricultural injuries, regardless of dementia status (Group 1 vs. Group 2). Given Group 1's small sample size, this finding may reflect limited statistical power rather than true equivalence. Indeed, in one sensitivity analysis (where EMS use was included as a covariate), Group 1 was slightly more likely to experience more severe injuries than Group 2, though this may simply reflect a chance finding. Moreover, because dementia is often underdiagnosed—more so among those in rural areas—some individuals experiencing both dementia and an agricultural injury may have been misclassified in Group 2. Similarly, individuals whose injuries were indirectly related to an older coworker's cognitive impairment may have also been included in Group 2. These factors could attenuate differences between Groups 1 and 2, biasing estimates toward the null. Further, even if there is truly no difference in injury severity between Groups 1 and 2, this is noteworthy, suggesting that individuals with diagnosed dementia who experience an agricultural injury remain involved in activities that are just as hazardous as those without dementia. This is important because, to date, research on dementia‐related safety has largely overlooked occupational settings.

Overall, our findings broadly underscore the need for tailored safety interventions for older adults in agricultural communities. Furthermore, given the increasing average age of US farm operators and prior evidence suggesting a link between agricultural occupational exposures and dementia, the prevalence of dementia in this population could increase. The findings of this paper—that individuals with both agricultural injuries and dementia sustain injuries that are possibly equally or more severe than those in Groups 2 and 3—may have implications for dementia‐related intervention development for agricultural populations. This is particularly relevant as existing dementia safety programs largely target residential settings and overlook agricultural hazards like livestock, heavy equipment, and firearms [29]. Meanwhile, most farm safety programs for older adults generally focus only on physical limitations [30]. Additionally, these programs offer limited support to family members of older agricultural workers—those often tasked with implementing safety modifications and recognizing when it is truly unsafe for an individual to be participating in agricultural activities. Yet rural informal dementia caregivers encounter multiple barriers to accessing education and support [31]. In recent years, a few programs and resources have emerged to address safety implications of cognitive impairment among older adults in agricultural communities [32, 33, 34]. Further research is needed to understand their feasibility, acceptability, utilization, and efficacy. This study also highlights the role clinicians could play in preventing injuries among dementia patients in agricultural settings. While safety counseling in routine office visits is recommended for preventing childhood farm injuries [35], guidance for older farmers is lacking.

There are multiple limitations to this study. First, there were a relatively small number of patients with both agricultural injuries and dementia as compared to Groups 2 and 3. Consequently, the model fit was limited in some estimations. This may be due to underdiagnosis of dementia in rural areas [15]. It may also reflect that individuals with dementia are less likely to engage in agricultural work. At the same time, to the extent that a documented dementia diagnosis reflects a more advanced stage of the disease, our findings provide an underestimation of the overall scope of injury patterns in this population. Second, we are unable to distinguish injuries sustained during farming activities from those occurring on the farm but unrelated to work. This limitation can influence future intervention design—though the small number of programs that currently do address this issue include both farm workers and agricultural residents with dementia [32, 34].

Third, although our models account for EMS use in sensitivity analyses—which tends to be higher in urban than rural areas [26]—we cannot fully separate the effects of agricultural injury from those of rurality. Rurality is a key confounder: individuals with agricultural injuries are more likely to reside in rural areas, where access to definitive care is often delayed and Level I and Level II trauma centers are less available [26, 36], factors that can influence both injury severity and ED discharge disposition. Nevertheless, our primary goal is to better understand overall injury outcomes among individuals with dementia who experience agricultural injuries, regardless of whether differences arise from the nature of the injury itself or from variations in response times or trauma center resources. As mentioned previously, a related concern is potential lack of generalizability if Level I and II trauma centers are overrepresented in the data. This is an important limitation but beyond the scope of our current work. Future studies should consider regional analyses to illuminate geographic variation in the capture of agricultural injuries.

Fourth, because we are unable to link patients with trauma centers, we cannot cluster standard errors at the trauma center level, even though some patients may be nested within each center. Fifth, due to data limitations—particularly the small size of Group 1—we were unable to stratify results for periods prior to 2020 versus 2020–2021 to assess the potential impact of the COVID‐19 pandemic. Future research with larger sample sizes should explore how the pandemic may have influenced injury severity outcomes. Sixth, we were unable to test the sensitivity of our results to alternative dementia definitions. Finally, given the multiple comparisons conducted, some statistically significant findings may reflect chance rather than true associations. Overall, the goal of this cross‐sectional analysis is not to establish causal relationships.

## 5 Conclusion

This study characterizes and provides an overview of the injury burden among individuals experiencing agricultural injuries with a documented dementia diagnosis. Our findings suggest that this group may be vulnerable to severe injuries. These results should be interpreted as descriptive and hypothesis‐generating. Causal claims about dementia increasing agricultural injury risk or severity require stronger designs with validated dementia assessment, population‐based sampling, and distinction between occupational and residential injuries. Future research should explore this population in greater detail and assess the needs of farm families with one or more members living with dementia.

## Author Contributions

*Concept and design*: Kanika Arora and Jonathan Davis. *Acquisition of subjects and/or data*: Jonathan Davis. *Analysis and interpretation of data*: All authors. *Preparation of manuscript*: All authors.

## Ethics Statement

This study used de‐identified data under a Data Use Agreement (DUA) that prohibits re‐identification. Therefore, it was not considered human subjects research. The DUA procedures were reviewed and approved by the University of Iowa IRB.

## Conflicts of Interest

The authors declare no conflicts to disclose.

## Supporting information

Supporting File

## Data Availability

The data that support the findings of this study are available from Committee on Trauma, American College of Surgeons. Restrictions apply to the availability of these data, which were used under license for this study. Data are available from the author(s) with the permission of Committee on Trauma, American College of Surgeons.
