# Supplementary material for: Agricultural Injuries With Dementia: Double Whammy?
Source: Am J Ind Med. 2026 Apr 16;69(7):575–82. doi: 10.1002/ajim.70082 (PMC13241857; doi:10.1002/ajim.70082)
Supplement: Supplementary file 1 — Supporting File [file AJIM-69-575-s001.docx]

**Supplementary Material**

**for**

**Agricultural Injuries with Dementia: Double Whammy?**

**Legend for Supplemental Material**

- Table S1: ICD-10 Codes used to identify dementia cases
- Table S2: ED Discharge Disposition and Injury Characteristics of Trauma Patients (≥ 60y) by Dementia Status and Presence of Agricultural Injury
- Table S3A: Average Marginal Effects for Injury Severity (Group 1 vs. Group 2)
- Table S3B: Average Marginal Effects for ICU/Surgery Post ED among Hospitalized (Group 1 vs. Group 2)
- Table S3C: Average Marginal Effects for Injury Severity (Group 1 vs. Group 3)
- Table S3D: Average Marginal Effects for ICU/Surgery Post ED among Hospitalized (Group 1 vs. Group 3)
- Table S3E: Average Marginal Effects for Injury Severity (Group 1 vs. Group 3) – Accounting for Injury Mechanisms
- Table S3F: Average Marginal Effects for ICU/Surgery Post ED among Hospitalized (Group 1 vs. Group 3) - Accounting for Injury Mechanisms
- Table S3G: Average Marginal Effects for Injury Severity (Group 1 vs. Group 3) – Among those with Fall Injuries Only
- Table S3H. Average Marginal Effects for ICU/Surgery Post ED among Hospitalized (Group 1 vs. Group 3) – Among those with Fall Injuries Only
- Table S4A: Sensitivity Test 1: Adjusted Predicted Probabilities of Minor, Moderate, and Major Injury (Group 1 vs. Group 2)
- Table S4B: Sensitivity Test 1: Average Marginal Effects for Injury Severity (Group 1 vs. Group 2)
- Table S4C: Sensitivity Test 1: Adjusted Predicted Probability of ICU/Surgery Post ED among Hospitalized (Group 1 vs. Group 2)
- Table S4D: Sensitivity Test 1: Average Marginal Effects for ICU/Surgery Post ED among Hospitalized (Group 1 vs. Group 2)
- Table S4E: Sensitivity Test 1: Adjusted Predicted Probabilities of Minor, Moderate, and Major Injury (Group 1 vs. Group 3)
- Table S4F: Sensitivity Test 1: Average Marginal Effects for Injury Severity (Group 1 vs. Group 3)
- Table S4G: Sensitivity Test 1: Adjusted Predicted Probability of ICU/Surgery Post ED among Hospitalized (Group 1 vs. Group 3)
- Table S4H: Sensitivity Test 1: Average Marginal Effects for ICU/Surgery Post ED among Hospitalized (Group 1 vs. Group 3)
- Table 5A: Sensitivity Test 2: Adjusted Predicted Probabilities of Minor, Moderate, and Major Injury (Group 1 vs. Group 3)
- Table 5B: Sensitivity Test 2: Average Marginal Effects for Injury Severity (Group 1 vs. Group 3)
- Table 5C: Sensitivity Test 2: Adjusted Predicted Probability of ICU/Surgery Post ED among Hospitalized (Group 1 vs. Group 3)
- Table 5D: Sensitivity Test 2: Average Marginal Effects for ICU/Surgery Post ED among Hospitalized (Group 1 vs. Group 3)
- Table S6A: Sensitivity Test 3: Average Marginal Effects for Injury Severity (Group 1 vs. Group 2) – Controlling for EMS use
- Table S6B. Sensitivity Test 3: Average Marginal Effects for ICU/Surgery Post ED among Hospitalized (Group 1 vs. Group 2) – Controlling for EMS use
- Table S6C. Sensitivity Test 3: Average Marginal Effects for Injury Severity (Group 1 vs. Group 3) – Controlling for EMS use
- Table S6D. Sensitivity Test 3: Average Marginal Effects for ICU/Surgery Post ED among Hospitalized (Group 1 vs. Group 3) – Controlling for EMS use

**Table S1**

| **ICD-10 Codes used to identify dementia cases**  F01.50, F01.51, F02.80, F02.81, F03.90, F03.91, F04, F05, F06.1, F06.8, F10.7, G13.8, G30.0, G30.1, G30.8, G30.9, G31.01, G31.09, G31.1, G31.2, G94, R41.81, R54 |
| --- |

| **Table S2. ED Discharge Disposition and Injury Characteristics of Trauma Patients (≥ 60 y) by Dementia Status and Presence of Agricultural Injury** | | | |  |
| --- | --- | --- | --- | --- |
|  | Dementia and Ag. Injury (Group 1) (*n*=318) | Only Ag. Injury (No Dementia) (Group 2) (*n*=21,361) | Only Dementia (No Ag. Injury) (Group 3) (*n*=231,231) |  |
|  |  |  |  |  |
| *ED Discharge Disposition (Col%)* |  |  |  |  |
| ***Not Hospitalized*** | ***6%*** | ***10%*** | ***6%*** |  |
|  |  |  |  |  |
| ***Hospitalized Post ED*** | ***89%*** | ***87%*** | ***91%*** |  |
| Floor bed/Observation/Telemetry | 53% | 53% | 68% |  |
| Intensive Care Unit/Operating Room (Surgery) | 36% | 34% | 23% |  |
|  |  |  |  |  |
| ***Direct Admit*** | ***6%*** | ***4%*** | ***4%*** |  |
| Direct admit | 6% | 4% | 4% |  |
|  |  |  |  |  |
| *Injury Severity Score (ISS) (Col %)* |  |  |  |  |
| ISS (standard deviation) | 10.91 (7.25) | 10.81 (7.86) | 9.23 (6.10) |  |
| Minor Injury (ISS=0-8) | 31% | 36% | 35% |  |
| Moderate Injury (ISS=9-15) | 50% | 43% | 51% |  |
| Major Injury (ISS>15) | 19% | 21% | 13% |  |
| *Notes: Data come from the 2017-2021 American College of Surgeons Trauma Quality Programs Participant Use File (ACS-TQP-PUF). “Not Hospitalized” includes deceased/expired, home with or without services, and left/transferred. Percentages for these sub-categories were similar across groups, except Group 2 had a slightly higher percentage (relative to Group 1 and Group 3) in home with or without services. Abbreviations: Ag., Agricultural; ED, Emergency Department* | | | |  |
|  |  |  |  |  |
|  |  |  |  |  |

| **TABLE S3A. Average Marginal Effects for Injury Severity (Group 1 vs. Group 2)** | | | | |  |
| --- | --- | --- | --- | --- | --- |
|  | dy/dx | | 95% CI; P Value | |  |
| Minor Injury | -0.03 | | [-0.09-0.02]; 0.27 | |  |
| Moderate Injury | 0.05 | | [-0.02-0.11]; 0.11 | |  |
| Major Injury | -0.02 | | [-0.06-0.03]; 0.47 | |  |
| Cragg-Uhler/Nagelkerke R2 | 0.014 | |  | |  |
| N | 21,668 | | | |  |
| *Notes*: Data come from the 2017-2021 American College of Surgeons Trauma Quality Programs Participant Use File (ACS-TQP-PUF). Sample includes Group 1 and Group 2. The table shows average marginal effects for minor, moderate, and major injury comparing Group 1 to Group 2. These estimates are based on a generalized ordered logit regression with controls for age categories, female, black, other, Hispanic, smoking status, comorbidities (diabetes, hypertension, Congestive Heart Failure, Myocardial Infarction, Chronic Obstructive Pulmonary Disorder), and year fixed effects. | | | | |  |
|  |  |  |  |  |  |
|  |  |  |  |  |  |
|  |  |  |  |  |  |
|  |  |  |  |  |  |
|  |  |  |  |  |  |
|  |  |  |  |  |  |
| **Table S3B. Average Marginal Effects for ICU/Surgery Post ED among Hospitalized (Group 1 vs. Group 2)** | | | | |  |
|  |  |  |  |  |  |
|  | dy/dx | | 95% CI; P Value | |  |
| ICU/Surgery Post-ED among Hospitalized | 0.003 | | [-0.05-0.06]; 0.92 | |  |
| Hosmer-Lemeshow chi2(8) | 2.84 | | | |  |
| Prob > chi 2 | 0.94 | | | |  |
| N | 18,839 | | | |  |
| *Notes*: Data come from the 2017-2021 American College of Surgeons Trauma Quality Programs Participant Use File (ACS-TQP-PUF). Sample (ED patients discharged to the hospital) includes Group 1 and Group 2. The table shows average marginal effects for ICU/Surgery Post ED among Hospitalized comparing Group 1 to Group 2. These estimates are based on a logistic regression that also controls for age categories, female, black, other, Hispanic, smoking status, comorbidities (diabetes, hypertension, Congestive Heart Failure, Myocardial Infarction, Chronic Obstructive Pulmonary Disorder), and year fixed effects. | | | | |  |
|  |  |  |  |  |  |
|  |  |  |  |  |  |
|  |  |  |  |  |  |

| **TABLE S3C. Average Marginal Effects for Injury Severity (Group 1 vs. Group 3)** | | | | |  |
| --- | --- | --- | --- | --- | --- |
|  | dy/dx | | 95% CI; P value | |  |
| Minor Injury | -0.05 | | [-0.10-0.005]; 0.07 | |  |
| Moderate Injury | 0.02 | | [-0.03-0.07]; 0.64 | |  |
| Major Injury | 0.03 | | [0.001-0.06]; 0.04 | |  |
| Cragg-Uhler/Nagelkerke R2 | 0.014 | |  | |  |
| N | 231,168 | | | |  |
| *Notes*: Data come from the 2017-2021 American College of Surgeons Trauma Quality Programs Participant Use File (ACS-TQP-PUF). Sample includes Group 1 and Group 3. The table shows average marginal effects for minor, moderate, and major injury comparing Group 1 to Group3. These estimates are based on a generalized ordered logit regression with controls for age categories, female, black, other, Hispanic, smoking status, comorbidities (diabetes, hypertension, Congestive Heart Failure, Myocardial Infarction, Chronic Obstructive Pulmonary Disorder), and year fixed effects. | | | | |  |
|  |  |  |  |  |  |
|  |  |  |  |  |  |
|  |  |  |  |  |  |
|  |  |  |  |  |  |
|  |  |  |  |  |  |
|  |  |  |  |  |  |
| **TABLE S3D. Average Marginal Effects for ICU/Surgery Post ED among Hospitalized (Group 1 vs. Group 3)** | | | | |  |
|  |  |  |  |  |  |
|  | dy/dx | | 95% CI; P Value | |  |
| ICU/Surgery Post-ED among Hospitalized | 0.12 | | [0.06-0.17]; <0.001 | |  |
| Hosmer-Lemeshow chi2(8) |  | | 13.83 | |  |
| Prob > chi 2 |  | | 0.09 | |  |
| N | 209,802 | | | |  |
| *Notes*: Data come from the 2017-2021 American College of Surgeons Trauma Quality Programs Participant Use File (ACS-TQP-PUF). Sample (ED patients discharged to the hospital) includes Group 1 and Group 3. The table shows average marginal effects for ICU/Surgery Post ED among Hospitalized comparing Group 1 to Group 3. These estimates are based on a logistic regression that also controls for age categories, female, black, other, Hispanic, smoking status, comorbidities (diabetes, hypertension, Congestive Heart Failure, Myocardial Infarction, Chronic Obstructive Pulmonary Disorder), and year fixed effects. | | | | |  |
|  |  |  |  |  |  |
|  |  |  |  |  |  |
|  |  |  |  |  |  |

| **TABLE S3E. Average Marginal Effects for Injury Severity (Group 1 vs. Group 3) – Accounting for Injury Mechanisms** | | | | |  |
| --- | --- | --- | --- | --- | --- |
|  | dy/dx | | 95% CI; P value | |  |
| Minor Injury | -0.08 | | [-0.14 - -0.02]; 0.01 | |  |
| Moderate Injury | 0.08 | | [0.02-0.14]; 0.005 | |  |
| Major Injury | -0.00 | | [-0.04-0.03]; 0.78 | |  |
| Cragg-Uhler/Nagelkerke R2 | 0.024 | |  | |  |
| N | 230,340 | | | |  |
| *Notes*: Data come from the 2017-2021 American College of Surgeons Trauma Quality Programs Participant Use File (ACS-TQP-PUF). Sample includes Group 1 and Group 3. The table shows average marginal effects for minor, moderate, and major injury comparing Group 1 to Group3. These estimates are based on a generalized ordered logit regression with controls for age categories, female, black, other, Hispanic, smoking status, comorbidities (diabetes, hypertension, Congestive Heart Failure, Myocardial Infarction, Chronic Obstructive Pulmonary Disorder), injury mechanism (falls, motor vehicle traffic, transport, machinery, environment, struck by/against) and year fixed effects. | | | | |  |
|  |  |  |  |  |  |
|  |  |  |  |  |  |
|  |  |  |  |  |  |
|  |  |  |  |  |  |
|  |  |  |  |  |  |
|  |  |  |  |  |  |
| **TABLE S3F. Average Marginal Effects for ICU/Surgery Post ED among Hospitalized (Group 1 vs. Group 3) - Accounting for Injury Mechanisms** | | | | |  |
|  |  |  |  |  |  |
|  | dy/dx | | 95% CI; P Value | |  |
| ICU/Surgery Post-ED among Hospitalized | 0.04 | | [-0.01-0.09]; 0.13 | |  |
| Hosmer-Lemeshow chi2(8) |  | | 23.43 | |  |
| Prob > chi 2 |  | | 0.003 | |  |
| N | 209,058 | | | |  |
| *Notes*: Data come from the 2017-2021 American College of Surgeons Trauma Quality Programs Participant Use File (ACS-TQP-PUF). Sample (ED patients discharged to the hospital) includes Group 1 and Group 3. The table shows average marginal effects for ICU/Surgery Post ED among Hospitalized comparing Group 1 to Group 3. These estimates are based on a logistic regression that also controls for age categories, female, black, other, Hispanic, smoking status, comorbidities (diabetes, hypertension, Congestive Heart Failure, Myocardial Infarction, Chronic Obstructive Pulmonary Disorder), injury mechanism (falls, motor vehicle traffic, transport, machinery, environment, struck by/against) and year fixed effects. | | | | |  |
|  |  |  |  |  |  |
|  |  |  |  |  |  |
|  |  |  |  |  |  |

| **TABLE S3G. Average Marginal Effects for Injury Severity (Group 1 vs. Group 3) – Among those with Fall Injuries Only** | | | | |  |
| --- | --- | --- | --- | --- | --- |
|  | dy/dx | | 95% CI; P value | |  |
| Minor Injury | -0.02 | | [-0.11 - 0.07]; 0.70 | |  |
| Moderate Injury | -0.01 | | [0.10 - 0.08]; 0.89 | |  |
| Major Injury | 0.02 | | [-0.03 - 0.08]; 0.39 | |  |
| Cragg-Uhler/Nagelkerke R2 | 0.013 | |  | |  |
| N | 217,420 | | | |  |
| *Notes*: Data come from the 2017-2021 American College of Surgeons Trauma Quality Programs Participant Use File (ACS-TQP-PUF). Sample includes members of Groups 1 and 3 injured due to a fall. The table shows average marginal effects for minor, moderate, and major injury comparing Group 1 to Group3. These estimates are based on a generalized ordered logit regression with controls for age categories, female, black, other, Hispanic, smoking status, comorbidities (diabetes, hypertension, Congestive Heart Failure, Myocardial Infarction, Chronic Obstructive Pulmonary Disorder), injury mechanism (falls, motor vehicle traffic, transport, machinery, environment, struck by/against) and year fixed effects. | | | | |  |
|  |  |  |  |  |  |
|  |  |  |  |  |  |
|  |  |  |  |  |  |
|  |  |  |  |  |  |
|  |  |  |  |  |  |
|  |  |  |  |  |  |
| **TABLE S3H. Average Marginal Effects for ICU/Surgery Post ED among Hospitalized (Group 1 vs. Group 3) – Among those with Fall Injuries Only** | | | | |  |
|  |  |  |  |  |  |
|  | dy/dx | | 95% CI; P Value | |  |
| ICU/Surgery Post-ED among Hospitalized | 0.02 | | [-0.06 - 0.11]; 0.63 | |  |
| Hosmer-Lemeshow chi2(8) |  | | 13.45 | |  |
| Prob > chi 2 |  | | 0.10 | |  |
| N | 197,404 | | | |  |
| *Notes*: Data come from the 2017-2021 American College of Surgeons Trauma Quality Programs Participant Use File (ACS-TQP-PUF). Sample (ED patients discharged to the hospital) includes Group 1 and Group 3 members injured due to a fall. The table shows average marginal effects for ICU/Surgery Post ED among Hospitalized comparing Group 1 to Group 3. These estimates are based on a logistic regression that also controls for age categories, female, black, other, Hispanic, smoking status, comorbidities (diabetes, hypertension, Congestive Heart Failure, Myocardial Infarction, Chronic Obstructive Pulmonary Disorder), injury mechanism (falls, motor vehicle traffic, transport, machinery, environment, struck by/against) and year fixed effects. | | | | |  |
|  |  |  |  |  |  |
|  |  |  |  |  |  |
|  |  |  |  |  |  |

| **TABLE S4A. Sensitivity Test 1: Adjusted Predicted Probabilities of Minor, Moderate, and Major Injury (Group 1 vs. Group 2)** | | |  |
| --- | --- | --- | --- |
|  | Dementia AND CONFIRMED Agricultural Injury (Group 1) | Only CONFIRMED Agricultural Injury (NO Dementia) (Group 2) |  |
| Probability of Minor Injury (ISS 1-8) | 0.34 | 0.36 |  |
| [95% CI] | [0.27-0.41] | [0.35-0.36] |  |
| Probability of Moderate Injury (ISS 9-15) | 0.44 | 0.43 |  |
| [95% CI] | [0.37-0.52] | [0.42-0.44] |  |
| Probability of Major Injury (ISS > 15) | 0.22 | 0.21 |  |
| [95% CI] | [0.16-0.28] | [0.20-0.22] |  |
| N | 12,978 | |  |
| *Notes*: Data come from the 2017-2021 American College of Surgeons Trauma Quality Programs Participant Use File (ACS-TQP-PUF). Sample includes revised versions of Group 1 and Group 2 that are restricted to confirmed agricultural injury cases. Table shows adjusted predicted probabilities for minor, moderate, and major injury. These estimates are based on a generalized ordered logit regression that controls for group (1 vs 2), age categories, female, black, other, Hispanic, smoking status, comorbidities (diabetes, hypertension, Congestive Heart Failure, Myocardial Infarction, Chronic Obstructive Pulmonary Disorder), and year fixed effects. | | |  |
|  |  |  |  |
|  |  |  |  |
|  |  |  |  |

| **TABLE S4B. Sensitivity Test 1: Average Marginal Effects for Injury Severity (Group 1 vs. Group 2)** | | |  |
| --- | --- | --- | --- |
|  |  |  |  |
|  | dy/dx | 95% CI; P Value |  |
| Minor Injury | -0.16 | [-0.08-0.06]; 0.65 |  |
| Moderate Injury | 0.13 | [-0.06-0.08]; 0.74 |  |
| Major Injury | 0.004 | [-0.06-0.06]; 0.90 |  |
| Cragg-Uhler/Nagelkerke R2 | 0.018 |  |  |
| N | 12,978 | |  |
| *Notes*: Data come from the 2017-2021 American College of Surgeons Trauma Quality Programs Participant Use File (ACS-TQP-PUF). Sample includes revised versions of Group 1 and Group 2 that are restricted to confirmed agricultural injury cases. The table shows average marginal effects for minor, moderate, and major injury comparing Group 1 to Group 2. These estimates are based on a generalized ordered logit regression with controls for age categories, female, black, other, Hispanic, smoking status, comorbidities (diabetes, hypertension, Congestive Heart Failure, Myocardial Infarction, Chronic Obstructive Pulmonary Disorder), and year fixed effects. | | |  |
|  |  |  |  |
|  |  |  |  |
|  |  |  |  |
|  |  |  |  |

| **TABLE S4C. Sensitivity Test 1: Adjusted Predicted Probability of ICU/Surgery Post ED among Hospitalized (Group 1 vs. Group 2)** | | |  |
| --- | --- | --- | --- |
|  | Dementia AND CONFIRMED Agricultural Injury (Group 1) | Only CONFIRMED Agricultural Injury (NO Dementia) (Group 2) |  |
| Probability of ICU/Surgery Post ED among Hospitalized | 0.39 | 0.40 |  |
| [95% CI] | [0.32-0.47] | [0.39-0.40] |  |
| N | 11,228 | |  |
| *Notes*: Data come from the 2017-2021 American College of Surgeons Trauma Quality Programs Participant Use File (ACS-TQP-PUF). Sample (ED patients discharged to the hospital) includes revised versions of Group 1 and Group 2 that are restricted to confirmed agricultural injury cases. The table shows adjusted predicted probabilities for ICU/Surgery Post ED among hospitalized patients. These estimates are based on a logistic regression that controls for group (1 vs 2), age categories, female, black, other, Hispanic, smoking status, comorbidities (diabetes, hypertension, Congestive Heart Failure, Myocardial Infarction, Chronic Obstructive Pulmonary Disorder), and year fixed effects. | | |  |
|  |  |  |  |
|  |  |  |  |
|  |  |  |  |

| **TABLE S4D. Sensitivity Test 1: Average Marginal Effects for ICU/Surgery Post ED among Hospitalized (Group 1 vs. Group 2)** | | |  |
| --- | --- | --- | --- |
|  |  |  |  |
|  | dy/dx | 95% CI; P Value |  |
| ICU/Surgery Post-ED among Hospitalized | -0.002 | [-0.07-0.07]; 0.95 |  |
| Hosmer-Lemeshow chi2(8) | 7.90 |  |  |
| Prob > chi 2 | 0.44 |  |  |
| N | 11,228 | |  |
| *Notes*: Data come from the 2017-2021 American College of Surgeons Trauma Quality Programs Participant Use File (ACS-TQP-PUF). Sample (ED patients discharged to the hospital) includes revised versions of Group 1 and Group 2 that are restricted to confirmed agricultural injury cases. The table shows average marginal effects for ICU/Surgery Post ED among Hospitalized comparing Group 1 to Group 2. These estimates are based on a logistic regression that also controls for age categories, female, black, other, Hispanic, smoking status, comorbidities (diabetes, hypertension, Congestive Heart Failure, Myocardial Infarction, Chronic Obstructive Pulmonary Disorder), and year fixed effects. | | |  |
|  |  |  |  |
|  |  |  |  |
|  |  |  |  |

| **TABLE S4E. Sensitivity Test 1: Adjusted Predicted Probabilities of Minor, Moderate, and Major Injury (Group 1 vs. Group 3)** | | |  |
| --- | --- | --- | --- |
|  | Dementia AND CONFIRMED Agricultural Injury (Group 1R) | Only Dementia (NO CONFIRMED Agricultural Injury) (Group 3R) |  |
| Probability of Minor Injury (ISS 1-8) | 0.31 | 0.35 |  |
| [95% CI] | [0.24-0.37] | [0.35-0.36] |  |
| Probability of Moderate Injury (ISS 9-15) | 0.51 | 0.52 |  |
| [95% CI] | [0.44-0.58] | [0.51-0.52] |  |
| Probability of Major Injury (ISS > 15) | 0.18 | 0.13 |  |
| [95% CI] | [0.13-0.23] | [0.12-0.13] |  |
| N | 231,168 | |  |
| *Notes*: Data come from the 2017-2021 American College of Surgeons Trauma Quality Programs Participant Use File (ACS-TQP-PUF). Sample includes revised versions of Group 1 and Group 3 that are restricted to confirmed agricultural injury cases. The table shows adjusted predicted probabilities for minor, moderate, and major injury. These estimates are based on a generalized ordered logit regression that controls for group (1 vs 3), age categories, female, black, other, Hispanic, smoking status, comorbidities (diabetes, hypertension, Congestive Heart Failure, Myocardial Infarction, Chronic Obstructive Pulmonary Disorder), and year fixed effects. | | |  |
|  |  |  |  |
|  |  |  |  |
|  |  |  |  |

| **TABLE S4F. Sensitivity Test 1: Average Marginal Effects for Injury Severity (Group 1 vs. Group 3)** | | |  |
| --- | --- | --- | --- |
|  |  |  |  |
|  | dy/dx | 95% CI; P Value |  |
| Minor Injury | -0.05 | [-0.12-0.02]; 0.18 |  |
| Moderate Injury | 0.002 | [-0.07-0.07]; 0.07 |  |
| Major Injury | 0.05 | [0.005-0.09]; 0.03 |  |
| Cragg-Uhler/Nagelkerke R2 | 0.014 |  |  |
| N | 231,168 | |  |
| *Notes*: Data come from the 2017-2021 American College of Surgeons Trauma Quality Programs Participant Use File (ACS-TQP-PUF). Sample includes revised versions of Group 1 and Group 3 that are restricted to confirmed agricultural injury cases. The table shows average marginal effects for minor, moderate, and major injury comparing Group 1 to Group 3. These estimates are based on a generalized ordered logit regression with controls for age categories, female, black, other, Hispanic, smoking status, comorbidities (diabetes, hypertension, Congestive Heart Failure, Myocardial Infarction, Chronic Obstructive Pulmonary Disorder), and year fixed effects. | | |  |
|  |  |  |  |
|  |  |  |  |
|  |  |  |  |
|  |  |  |  |

| **TABLE S4G. Sensitivity Test 1: Adjusted Predicted Probability of ICU/Surgery Post ED among Hospitalized (Group 1 vs. Group 3)** | | |  |
| --- | --- | --- | --- |
|  | Dementia AND CONFIRMED Agricultural Injury (Group 1) | Only Dementia (NO CONFIRMED Agricultural Injury) (Group 3) |  |
| Probability of ICU/Surgery Post ED among Hospitalized | 0.36 | 0.25 |  |
| [95% CI] | [0.29-0.44] | [0.24-0.25] |  |
| N | 209,802 | |  |
| *Notes*: Data come from the 2017-2021 American College of Surgeons Trauma Quality Programs Participant Use File (ACS-TQP-PUF). Sample (ED patients discharged to the hospital) includes revised versions of Group 1 and Group 3 that are restricted to confirmed agricultural injury cases. Table shows adjusted predicted probabilities for ICU/Surgery Post ED among hospitalized patients. These estimates are based on a logistic regression that controls for group (1 vs 3), age categories, female, black, other, Hispanic, smoking status, comorbidities (diabetes, hypertension, Congestive Heart Failure, Myocardial Infarction, Chronic Obstructive Pulmonary Disorder), and year fixed effects. | | |  |
|  |  |  |  |
|  |  |  |  |
|  |  |  |  |

| **TABLE S4H. Sensitivity Test 1: Average Marginal Effects for ICU/Surgery Post ED among Hospitalized (Group 1 vs. Group 3)** | | |  |
| --- | --- | --- | --- |
|  |  |  |  |
|  | dy/dx | 95% CI; P Value |  |
| ICU/Surgery Post-ED among Hospitalized | 0.11 | [0.04-0.18]; 0.002 |  |
| Hosmer-Lemeshow chi2(8) | 14.12 |  |  |
| Prob > chi 2 | 0.08 |  |  |
| N | 209,802 | |  |
| *Notes*: Data come from the 2017-2021 American College of Surgeons Trauma Quality Programs Participant Use File (ACS-TQP-PUF). Sample (ED patients discharged to the hospital) includes revised versions of Group 1 and Group 3 that are restricted to confirmed agricultural injury cases. The table shows average marginal effects for ICU/Surgery Post ED among Hospitalized comparing Group 1 to Group 3. These estimates are based on a logistic regression that also controls for age categories, female, black, other, Hispanic, smoking status, comorbidities (diabetes, hypertension, Congestive Heart Failure, Myocardial Infarction, Chronic Obstructive Pulmonary Disorder), and year fixed effects. | | |  |
|  |  |  |  |
|  |  |  |  |
|  |  |  |  |

| **TABLE S5A. Sensitivity Test 2: Adjusted Predicted Probabilities of Minor, Moderate, and Major Injury (Group 1 vs. Group 3)** | | |  |
| --- | --- | --- | --- |
|  | Dementia and Ag. Injury (Group 1) (n=318) | Only Dementia (No Ag. Or Residential Injury) (Group 3) (n=28,571) |  |
| Probability of Minor Injury (ISS 1-8) | 0.30 | 0.37 |  |
| [95% CI] | [0.26-0.36] | [0.36-0.38] |  |
| Probability of Moderate Injury (ISS 9-15) | 0.51 | 0.43 |  |
| [95% CI] | [0.45-0.56] | [0.43-0.44] |  |
| Probability of Major Injury (ISS > 15) | 0.18 | 0.19 |  |
| [95% CI] | [0.14-0.22] | [0.18-0.19] |  |
| N | 28,848 | |  |
| *Notes*: Data come from the 2017-2021 American College of Surgeons Trauma Quality Programs Participant Use File (ACS-TQP-PUF). Sample includes Group 1 and Group 3 where Group 3 excludes agricultural and residential injuries. The table shows adjusted predicted probabilities for minor, moderate, and major injury. These estimates are based on a generalized ordered logit regression that controls for group (1 vs 3), age categories, female, black, other, Hispanic, smoking status, comorbidities (diabetes, hypertension, Congestive Heart Failure, Myocardial Infarction, Chronic Obstructive Pulmonary Disorder), and year fixed effects. | | |  |
|  |  |  |  |
|  |  |  |  |
|  |  |  |  |

| **TABLE S5B. Sensitivity Test 2: Average Marginal Effects for Injury Severity (Group 1 vs. Group 3)** | | |  |
| --- | --- | --- | --- |
|  |  |  |  |
|  | dy/dx | 95% CI; P Value |  |
| Minor Injury | -0.07 | [-0.12 - -0.01]; 0.01 |  |
| Moderate Injury | 0.08 | [0.02 - 0.13]; 0.01 |  |
| Major Injury | -0.01 | [-0.05 - 0.04]; 0.79 |  |
| Cragg-Uhler/Nagelkerke R2 | 0.011 |  |  |
| N | 28,848 | |  |
| *Notes*: Data come from the 2017-2021 American College of Surgeons Trauma Quality Programs Participant Use File (ACS-TQP-PUF). Sample includes Group 1 and Group 3 where Group 3 excludes agricultural and residential injuries. The table shows average marginal effects for minor, moderate, and major injury comparing Group 1 to Group 3. These estimates are based on a generalized ordered logit regression with controls for age categories, female, black, other, Hispanic, smoking status, comorbidities (diabetes, hypertension, Congestive Heart Failure, Myocardial Infarction, Chronic Obstructive Pulmonary Disorder), and year fixed effects. | | |  |
|  |  |  |  |
|  |  |  |  |
|  |  |  |  |
|  |  |  |  |

| **TABLE S5C. Sensitivity Test 2: Adjusted Predicted Probability of ICU/Surgery Post ED among Hospitalized (Group 1 vs. Group 3)** | | |  |
| --- | --- | --- | --- |
|  | Dementia and Ag. Injury  (Group 1) (n=318) | Only Dementia (No Ag. Or Residential Injury) (Group 3) (n=28,571) |  |
| Probability of ICU/Surgery Post ED among Hospitalized | 0.38 | 0.34 |  |
| [95% CI] | [0.33-0.44] | [0.33-0.34] |  |
| N | 25,833 | |  |
| *Notes*: Data come from the 2017-2021 American College of Surgeons Trauma Quality Programs Participant Use File (ACS-TQP-PUF). Sample (ED patients discharged to the hospital) includes Group 1 and Group 3 where Group 3 excludes residential injuries. The table shows adjusted predicted probabilities for ICU/Surgery Post ED among hospitalized patients. These estimates are based on a logistic regression that controls for group (1 vs 3), age categories, female, black, other, Hispanic, smoking status, comorbidities (diabetes, hypertension, Congestive Heart Failure, Myocardial Infarction, Chronic Obstructive Pulmonary Disorder), and year fixed effects. | | |  |
|  |  |  |  |
|  |  |  |  |
|  |  |  |  |

| **TABLE S5D. Sensitivity Test 2: Average Marginal Effects for ICU/Surgery Post ED among Hospitalized (Group 1 vs. Group 3)** | | |  |
| --- | --- | --- | --- |
|  |  |  |  |
|  | dy/dx | 95% CI; P Value |  |
| ICU/Surgery Post-ED among Hospitalized | 0.05 | [-0.01-0.10]; 0.09 |  |
| Hosmer-Lemeshow chi2(8) | 7.08 |  |  |
| Prob > chi 2 | 0.53 |  |  |
| N | 25,833 | |  |
| *Notes*: Data come from the 2017-2021 American College of Surgeons Trauma Quality Programs Participant Use File (ACS-TQP-PUF). Sample (ED patients discharged to the hospital) includes Group 1 and Group 3 where Group 3 excludes residential injuries. The table shows average marginal effects for ICU/Surgery Post ED among Hospitalized comparing Group 1 to Group 3. These estimates are based on a logistic regression that also controls for age categories, female, black, other, Hispanic, smoking status, comorbidities (diabetes, hypertension, Congestive Heart Failure, Myocardial Infarction, Chronic Obstructive Pulmonary Disorder), and year fixed effects. | | |  |
|  |  |  |  |
|  |  |  |  |
|  |  |  |  |

| **TABLE S6A. Sensitivity Test 3: Average Marginal Effects for Injury Severity (Group 1 vs. Group 2) – Controlling for EMS use** | | | | |
| --- | --- | --- | --- | --- |
|  | dy/dx | | 95% CI; P Value | |
| Minor Injury | -0.03 | | [-0.09-0.02]; 0.24 | |
| Moderate Injury | 0.05 | | [-0.00-0.11]; 0.09 | |
| Major Injury | -0.02 | | [-0.06-0.03]; 0.45 | |
| Cragg-Uhler/Nagelkerke R2 | 0.05 | |  | |
| N | 21,567 | | | |
| *Notes*: Data come from the 2017-2021 American College of Surgeons Trauma Quality Programs Participant Use File (ACS-TQP-PUF). Sample includes Group 1 and Group 2. The table shows average marginal effects for minor, moderate, and major injury comparing Group 1 to Group 2. These estimates are based on a generalized ordered logit regression with controls for age categories, female, black, other, Hispanic, smoking status, comorbidities (diabetes, hypertension, Congestive Heart Failure, Myocardial Infarction, Chronic Obstructive Pulmonary Disorder), use of EMS transport and year fixed effects. | | | | |
|  |  |  |  |  |
|  |  |  |  |  |
|  |  |  |  |  |
|  |  |  |  |  |
|  |  |  |  |  |
|  |  |  |  |  |
| **Table S6B. Sensitivity Test 3: Average Marginal Effects for ICU/Surgery Post ED among Hospitalized (Group 1 vs. Group 2) – Controlling for EMS use** | | | | |
|  |  |  |  |  |
|  | dy/dx | | 95% CI; P Value | |
| ICU/Surgery Post-ED among Hospitalized | 0.003 | | [-0.05-0.06]; 0.09 | |
| Hosmer-Lemeshow chi2(8) | 7.24 | | | |
| Prob > chi 2 | 0.51 | | | |
| N | 18,761 | | | |
| *Notes*: Data come from the 2017-2021 American College of Surgeons Trauma Quality Programs Participant Use File (ACS-TQP-PUF). Sample (ED patients discharged to the hospital) includes Group 1 and Group 2. The table shows average marginal effects for ICU/Surgery Post ED among Hospitalized comparing Group 1 to Group 2. These estimates are based on a logistic regression that also controls for age categories, female, black, other, Hispanic, smoking status, comorbidities (diabetes, hypertension, Congestive Heart Failure, Myocardial Infarction, Chronic Obstructive Pulmonary Disorder), use of EMS transport, and year fixed effects. | | | | |
|  |  |  |  |  |
|  |  |  |  |  |
|  |  |  |  |  |

| **TABLE S6C. Sensitivity Test 3: Average Marginal Effects for Injury Severity (Group 1 vs. Group 3) – Controlling for EMS use** | | | | |  |
| --- | --- | --- | --- | --- | --- |
|  | dy/dx | | 95% CI; P value | |  |
| Minor Injury | -0.06 | | [-0.11- -0.005]; 0.03 | |  |
| Moderate Injury | 0.02 | | [-0.03-0.08]; 0.89 | |  |
| Major Injury | 0.03 | | [0.003-0.07]; 0.03 | |  |
| Cragg-Uhler/Nagelkerke R2 | 0.02 | |  | |  |
| N | 230,175 | | | |  |
| *Notes*: Data come from the 2017-2021 American College of Surgeons Trauma Quality Programs Participant Use File (ACS-TQP-PUF). Sample includes Group 1 and Group 3. The table shows average marginal effects for minor, moderate, and major injury comparing Group 1 to Group3. These estimates are based on a generalized ordered logit regression with controls for age categories, female, black, other, Hispanic, smoking status, comorbidities (diabetes, hypertension, Congestive Heart Failure, Myocardial Infarction, Chronic Obstructive Pulmonary Disorder), use of EMS transport, and year fixed effects. | | | | |  |
|  |  |  |  |  |  |
|  |  |  |  |  |  |
|  |  |  |  |  |  |
|  |  |  |  |  |  |
|  |  |  |  |  |  |
|  |  |  |  |  |  |
| **TABLE S6D. Sensitivity Test 3: Average Marginal Effects for ICU/Surgery Post ED among Hospitalized (Group 1 vs. Group 3) – Controlling for EMS use** | | | | |  |
|  |  |  |  |  |  |
|  | dy/dx | | 95% CI; P Value | |  |
| ICU/Surgery Post-ED among Hospitalized | 0.12 | | [0.06-0.18]; <0.001 | |  |
| Hosmer-Lemeshow chi2(8) |  | | 31.52 | |  |
| Prob > chi 2 |  | | 0.00 | |  |
| N | 208,986 | | | |  |
| *Notes*: Data come from the 2017-2021 American College of Surgeons Trauma Quality Programs Participant Use File (ACS-TQP-PUF). Sample (ED patients discharged to the hospital) includes Group 1 and Group 3. The table shows average marginal effects for ICU/Surgery Post ED among Hospitalized comparing Group 1 to Group 3. These estimates are based on a logistic regression that also controls for age categories, female, black, other, Hispanic, smoking status, comorbidities (diabetes, hypertension, Congestive Heart Failure, Myocardial Infarction, Chronic Obstructive Pulmonary Disorder), use of EMS transport, and year fixed effects. | | | | |  |
|  |  |  |  |  |  |
|  |  |  |  |  |  |
|  |  |  |  |  |  |
